# Antarctic Penguins as Reservoirs of Diversity for Avian Avulaviruses

**DOI:** 10.1128/JVI.00271-19

**Published:** 2019-05-15

**Authors:** Michelle Wille, Malet Aban, Jing Wang, Nicole Moore, Songhua Shan, John Marshall, Daniel González-Acuña, Dhanasekaran Vijaykrishna, Jeff Butler, Jianning Wang, Richard J. Hall, David T. Williams, Aeron C. Hurt

**Affiliations:** a WHO Collaborating Centre for Reference and Research on Influenza, The Peter Doherty Institute for Infection and Immunity, Melbourne, Australia; b Institute of Environmental Science and Research Ltd., National Centre for Biosecurity and Infectious Diseases, Upper Hutt, New Zealand; c CSIRO Australian Animal Health Laboratory, Geelong, Victoria, Australia; d Victorian Infectious Disease Reference Laboratory, The Peter Doherty Institute for Infection and Immunity, Melbourne, Australia; e Facultad de Ciencias Veterinarias, Universidad de Concepción, Chillán, Chile; f Facultad de Ecología y Recursos Naturales, Universidad Andrés Bello, Santiago, Chile; g Biomedicine Discovery Institute and Department of Microbiology, Monash University, Melbourne, Victoria, Australia; St. Jude Children’s Research Hospital

**Keywords:** Adelie penguin, Antarctica, avian avulavirus, avian paramyxovirus, disease ecology, penguin, Sphenisciformes

## Abstract

Wild birds harbor a huge diversity of avian avulaviruses (formerly avian paramyxoviruses). Antarctic penguin species have been screened for avian avulaviruses since the 1980s and, as such, are known hosts of these viruses. In this study, we screened three penguin species from the South Shetland Islands and the Antarctic Peninsula for avian avulaviruses. We show that Adelie penguins (*Pygoscelis adeliae*) are hosts for four different avian avulavirus species, the recently described avian avulaviruses 17 to 19 and avian avulavirus 10-like, never before isolated in Antarctica. A total of 24 viruses were isolated and sequenced; avian avulavirus 17 was the most common, and phylogenetic analysis demonstrated patterns of occurrence, with different genetic clusters corresponding to penguin age and location. Following infection in specific-pathogen-free (SPF) chickens, all four avian avulavirus species were shed from the oral cavity for up to 7 days postinfection. There was limited shedding from the cloaca in a proportion of infected chickens, and all but one bird seroconverted by day 21. No clinical signs were observed. Taken together, we propose that penguin species, including Antarctic penguins, may be the central reservoir for a diversity of avian avulavirus species and that these viruses have the potential to infect other avian hosts.

**IMPORTANCE** Approximately 99% of all viruses are still to be described, and in our changing world, any one of these unknown viruses could potentially expand their host range and cause epidemic disease in wildlife, agricultural animals, or humans. Avian avulavirus 1 causes outbreaks in wild birds and poultry and is thus well described. However, for many avulavirus species, only a single specimen has been described, and their viral ecology and epidemiology are unknown. Through the detection of avian avulaviruses in penguins from Antarctica, we have been able to expand upon our understanding of three avian avulavirus species (avian avulaviruses 17 to 19) and report a potentially novel avulavirus species. Importantly, we show that penguins appear to play a key role in the epidemiology of avian avulaviruses, and we encourage additional sampling of this avian group.

## Abstract (precis)

Approximately 99% of all viruses are still to be described, and in our changing world, any one of these unknown viruses could potentially expand their host range and cause epidemic disease in wildlife, agricultural animals, or humans. Avian avulavirus 1 causes outbreaks in wild birds and poultry and is thus well described. However, for many avulavirus species, only a single specimen has been described, and their viral ecology and epidemiology are unknown. Through the detection of avian avulaviruses in penguins from Antarctica, we have been able to expand upon our understanding of three avian avulavirus species (avian avulaviruses 17 to 19) and report a potentially novel avulavirus species. Importantly, we show that penguins appear to play a key role in the epidemiology of avian avulaviruses, and we encourage additional sampling of this avian group.

## INTRODUCTION

Wild birds harbor a huge diversity of avian avulaviruses (AAvV), formally designated avian paramyxoviruses (1). There are currently 19 species, corresponding to avian paramyxoviruses 1 to 13, in addition to 6 novel species proposed since 2017 which have recently been ratified by the ICTV (2–5). These viruses are nonsegmented, single stranded, and have a negative-sense RNA genome which is approximately 15 kb long and contains six genes (6). Avian avulavirus 1 (Newcastle disease virus) is one of the most important viruses infecting avian species around the globe, resulting in substantial socioeconomic losses to the poultry industry (7–9). Despite the importance of this virus, little is known about the ecology of AAvV-1 in wild birds, which are important reservoirs of lentogenic strains (10, 11). Even less is known about the ecology of the other 18 AAvVs in wild birds, and for many, we have no understanding of host range or spatial and temporal patterns. However it is known that some of these viral species do cause mortality in chickens while maintaining a lentogenic phenotype in wild birds (12). Indeed, most studies on AAvVs in wild birds are *ad hoc*, with many detections occurring during studies on avian influenza A virus, when an unknown agglutinating agent is discovered (e.g., see references 10 and 13). As such, with the exception of AAvV-1, this viral genus remains largely unexplored because it does not cause significant signs of disease in the reservoir hosts and is not considered socioeconomically important.

Avian avulaviruses have been identified in over 240 avian species across 50 avian orders (8). The detection of AAvVs in Antarctica dates back to about the 1980s, with particular interest in the charismatic Sphenisciformes (penguins). Avian avulaviruses have been isolated from king penguins (*Aptenodytes patagonicus*), royal penguins (*Eudyptes schlegeli*), Adelie penguins (*Pygoscelis adeliae*), and gentoo penguins (*Pygoscelis papua*) from Antarctica (5, 14–18) and from little blue penguins (*Eudyptula minor*) (19) and rockhopper penguins (*Eudyptes chrysocome*) (20) from Australia and the Falkland Islands, respectively. Serology studies have revealed the presence of antibodies against AAvV-1 and unclassified isolates from the 1980s in Antarctic penguins (16, 18, 21), AAvV-1 and AAvV-8 in sub-Antarctic penguins (15, 20, 22), and AAvV-1, AAvV-2, and AAvV-3 in penguins in South America and Australia (19, 23). Three distinct AAvV species were isolated in the 1980s (isolates PV1 to -8, 78/179); however, these viruses were not described within the current taxonomic scheme and, therefore, remain obscure (14). Regardless, more recent sampling efforts have identified substantial diversity with the detection and/or isolation of the previously described AAvV-1, AAvV-2, and AAvV-10 and three novel AAvVs (17–19) in penguins from Antarctica, the sub-Antarctic, and South America (5, 20, 21, 24, 25).

In this study, we aimed to detect and characterize AAvVs in penguins in the South Shetland Islands and Antarctic Peninsula using genetic and antigenic techniques to provide insights into the ecology of these viruses. We demonstrate the presence of 4 different avian avulavirus species: three that have been previously described in Antarctic penguins and one distantly related to AAvV-10 but previously described in penguins in the Falkland Islands and Brazil. We also undertook *in vivo* experimental infections of chickens to assess the pathogenic potential of representative isolates. Overall, we demonstrate that penguins are an important reservoir for multiple species and genetic lineages of AAvVs.

## RESULTS

### Prevalence of avian avulaviruses in Adelie penguins.

**FIG 1:**
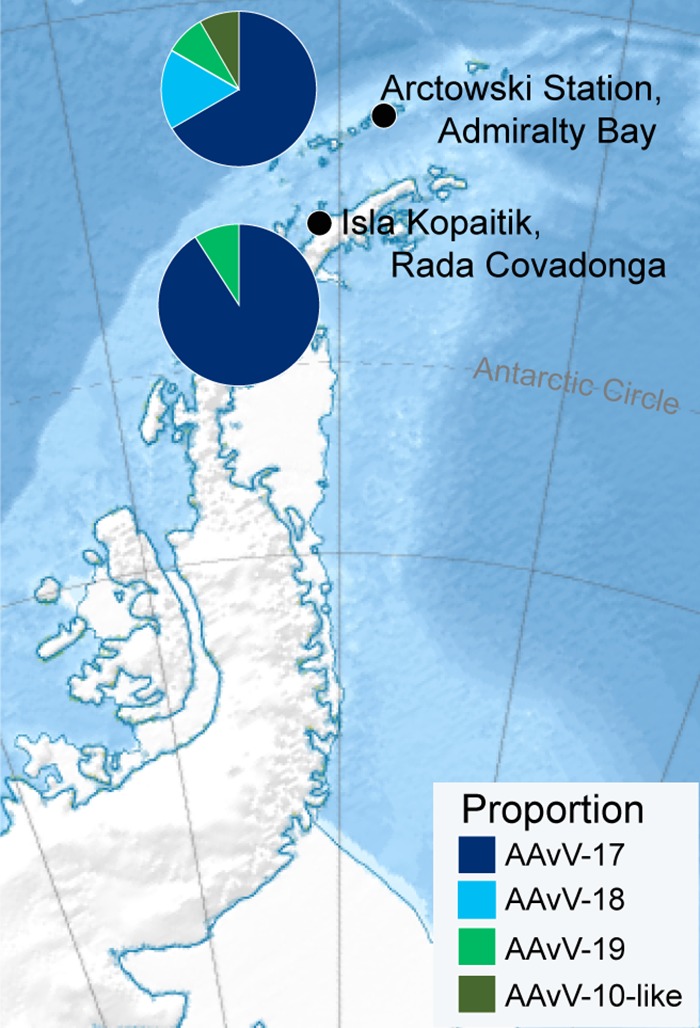
Antarctic sampling locations. Approximately equivalent numbers of Adelie penguins were sampled adjacent to Arctowski Station, Admiralty Bay, on King George Island and on Isla Kopaitik, adjacent to O’Higgens Base, Rada Covadonga. Chinstrap and gentoo penguins were also sampled at Isla Kopaitik. Pie charts depict the differences in proportions and occurrence of the four AAvVs identified in this study. The sizes of the pie charts are proportional to the number of isolates retrieved at each location. The relief map was sourced from Wikipedia, developed by user Kikos, and is distributed under a CC-by-SA 3.0 attribution.

Samples were obtained from 301 Adelie penguins from the south Shetland Islands (King George Island; 110 adults and 40 chicks) and Antarctic Peninsula (Isla Kopaitik, Rada Covadonga; 111 adults and 40 chicks) in 2013 (Fig. 1). Samples were also taken from a small number of gentoo penguins (*n* = 74) and chinstrap penguins (*Pygoscelis antarcticus*) (*n* = 18) from Isla Kopaitik.

Initial two-step reverse transcription-PCR screening attempts were not successful in detection of AAvVs; however, agglutinating agents were recovered using egg isolation and confirmed as AAvV through full-genome sequencing. Twenty-three AAvVs were isolated from Adelie penguins, and a single isolate (AAvV-19/chinstrap penguin/Antarctica/661/2013) was recovered from an adult chinstrap penguin at Rada Covadonga (Table 1). In Adelie penguins, the overall prevalence of AAvV was 7%, with chicks having a significantly higher prevalence (25%) than adults (1.357%) (*X*^2^ = 46.372, degree of freedom [df] = 1, *P* < 0.0001), but prevalence did not differ by location (*X*^2^ = 0.068, df = 1, *P* = 0.7937) (Table 1).

**TABLE 1:** Prevalence of avian avulaviruses in penguins sampled in Antarctica, 2013

Location	Age	Penguin species	No. of samples	% AAvV prevalence (95% CI)^*a*^	No. of isolates (% prevalence)			
					AAvV-17	AAvV-18	AAvV-19	AAvV-10-like
Admiralty Bay	Adult	Adelie	110	<0.001	0	0	0	0
	Chick	Adelie	40	30 (18–45)	8 (20)	2 (5)	1 (2.5 )	1 (2.5)
Rada Covadonga	Adult	Adelie	111	2.7 (0.9–7.6)	2 (1.8)	0	1(0.9)	0
	Chick	Adelie	40	20 (10–35)	8 (20)	0	0	0
	Adult	Gentoo	10	<0.001	0	0	0	0
	Chick	Gentoo	64	<0.001	0	0	0	0
	Adult	Chinstrap	18	5.56 (1–26)	0	0	1 (5.55)	0
Total			393		18 (6)	2 (0.7)	3 (0.7)	1 (0.3)

a Viruses were screened by isolation in embryonated chicken eggs to determine prevalence. 95% CI, 95% confidence interval.

### Patterns of evolutionary genetics of four AAvVs.

All 24 AAvV isolates identified in this study were sequenced. A total of 18,326,020 sequence reads were produced for the 24 samples using the Illumina MiSeq, with an average of 763,584 sequence reads per sample.

**FIG 2:**
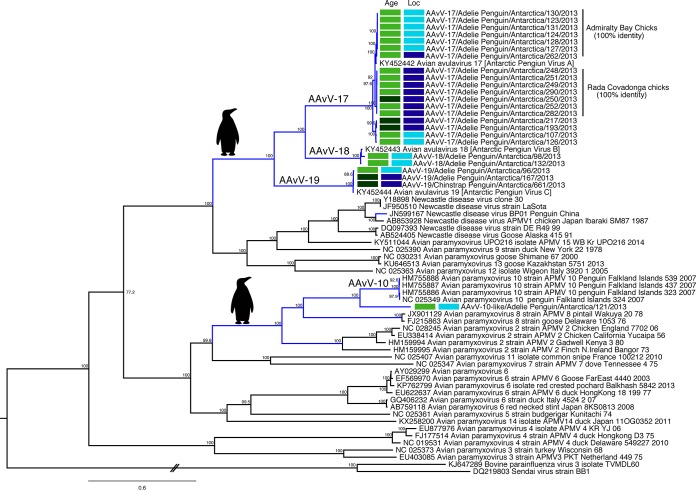
Maximum-likelihood tree for the full genomes of avian avulaviruses. Strain names from GenBank have been retained and, as such, reflect old taxonomic nomenclature. More than one reference virus for each species has been included (if available) to demonstrate interspecies diversity. Viruses isolated in this study are denoted by their designations and paired with metadata, displayed as colored boxes. The first box indicates the age (light green, chick; dark green, adult), and the second the location (light blue, Admiralty Bay; dark blue, Rada Covadonga). Penguin pictograms are placed at the most recent common ancestors (MRCA) of entire lineages which have been detected in penguins, and branches are colored blue if corresponding to a penguin virus in this tree. No penguin viruses for AAvV/avian paramyxovirus 2 (APMV-2) and AAvV/APMV-8 are shown in this tree; AAvV/APMV-2 has been isolated from penguins and only partially sequenced, as shown in Fig. S7 in the supplemental material, and AAvV/APMV-8 antibodies have been detected by HI. Viruses described by Neira et al. (5) as Antarctic Penguin Viruses A to C have recently been designated avian avulaviruses 17 to 19. Bovine parainfluenza virus and Sendai virus are set as the outgroup. The scale bar represents the number of nucleotide substitutions per site. Individual gene trees are presented in Fig. S1 to S6. The phylogeny of the partial L gene containing additional penguin sequences is presented in Fig. S7.

Phylogenetic analysis demonstrated that each of the 24 isolates belonged to one of four different AAvV species (Fig. 2). Phylogenetic congruence between the individual gene trees (Fig. 2, Fig. S1 to S7 in the supplemental material) suggests a lack of evidence for recombination between the penguin avulavirus lineages.

AAvV-17 and -19 were found in both locations, whereas AAvV-18 and AAvV-10-like were found only in Admiralty Bay (Table 1, Fig. 1). Interestingly, all four species were detected in chicks in Admiralty Bay during the sampling period. However, most of the viruses isolated were AAvV-17, with a prevalence of up to 20% in the Adelie penguin chicks in both Admiralty Bay and Rada Covadonga (Table 1, Fig. 1 and 2). The genetic similarity of AAvV-17 was 97.8%, and the isolates formed three distinct clades defined by location and penguin age. One clade was made up of viruses found almost exclusively in chicks from Admiralty Bay, one clade was viruses from chicks at Rada Covadonga, and the third clade of viruses was found in both locations and age groups, suggesting rapid spread in chicks following introduction in each of these locations. Indeed, the full-genome genetic similarity within each clade was >99%, and the clades comprising viruses from either the chicks from Admiralty Bay or the chicks from Rada Covadonga had viruses with 100% similarity (Fig. 2).

Avian avulaviruses 18 and 19 were less prevalent. However, while genetic diversity was low in AAvV-18 (99.2% identity), it was higher for AAvV-19 (97.7% identity), comparable to the variation seen among AAvV-17. Avian avulavirus 18 was found only in chicks from Admiralty Bay, in contrast to AAvV-19, which had high host and location heterogeneity, as it was isolated from an Adelie penguin chick from Admiralty Bay, adult Adelie penguins from Rada Covadonga, and an adult chinstrap penguin from Rada Covadonga (Table 1, Fig. 2). Finally, AAvV-10-like/Adelie penguin/Antarctica/121/2013 virus did not fall into this large penguin dominated clade consisting of AAvV-17, -18, and -19, but rather, was most closely related to AAvV-10, which has previously been isolated from penguins on the Falkland Islands and in Brazil (Fig. 2, Fig. S7).

### A novel avian avulavirus in Antarctic penguins?

Genetically and antigenically, it is unclear whether AAvV-10-like/Adelie penguin/Antarctica/121/2013 represents a novel AAvV species or whether it should be classified as a divergent AAvV-10 virus. This isolate and the AAvV-10 isolates from the Falkland Islands have a pairwise nucleotide similarity of 68.1% across the full genome and an L (RNA-dependent RNA polymerase) gene amino acid pairwise similarity of 85.1%. The nucleotide and amino acid pairwise similarities are marginally within the viral species classification threshold for nucleotide (60%) and amino acid (80%) pairwise similarity, respectively (26). To further resolve this, we assessed antigenicity using a hemagglutination inhibition (HI) assay, with a range of reference antigens and paired antisera. We included all four viral species detected in this study and reference antigens/antisera of AAvV-1 to -4, -6 to -10, and -12.

**FIG 3:**
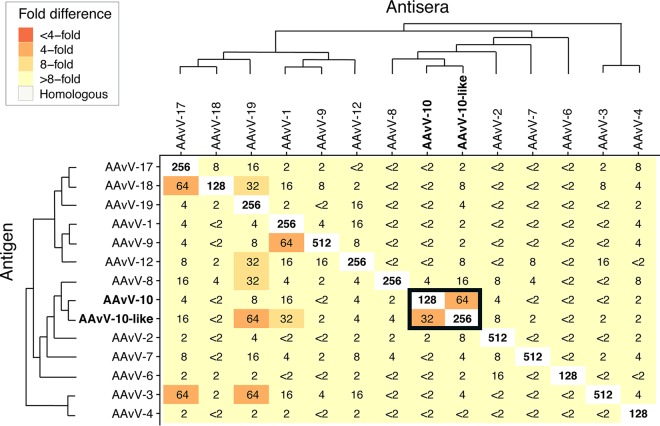
Results of hemagglutination inhibition (HI) assay demonstrating fold differences between reference viruses and four isolates recovered in this study, representing four different avian avulavirus species (AAvV-17, AAvV-18, AAvV-19, and AAvV-10-like). Viral species have been plotted in phylogenetic order, with a cladogram depicting phylogenetic relationships. HI titers are shown, and fold differences from the homologous titer are shown as a colored heatmap. The homologous antigen-antiserum titer is in boldface. Importantly, AAvV-10 and AAvV-10-like/Adelie penguin/Antarctica/121/2013, outlined by a black box, are 4-fold different, regardless of the antigen-antiserum combination. Viral species abbreviations are presented using the new nomenclature, i.e., AAvV rather than APMV. Antigens used in this study include APMV-1/chicken/Queensland/V4/66, APMV-2/chicken/California/Yucaipa/56, APMV-3/turkey/England/1087/82, APMV-4/chicken/WA/5030/84, APMV-6/duck/WA/694/78, APMV-7/dove/Tennessee/4/75, APMV-8/goose/Delaware/1053/75, APMV-9/duck/New York/22/78, APMV-10/Falkland Islands/penguin/EC324/2007, and APMV-12/wigeon/Italy/3920_1/05.

Compared to the homologous HI titer derived for the AAvV-10 reference antigen/antiserum (titer = 128), the AAvV-10 reference antiserum had reduced recognition for the AAvV-10-like/Adelie penguin/Antarctica/121/2013 virus, with an HI titer that was 4-fold lower (titer = 32) (Fig. 3). A similar 4-fold difference was observed using antiserum raised to the AAvV-10-like/Adelie penguin/Antarctica/121/2013 virus, which reacted strongly with its homologous virus but at 4-fold-lower levels against the reference AAvV-10 virus. The AAvV-10-like/Adelie penguin/Antarctica/121/2013 antigen also showed some reactivity with antiserum raised to AAvV-19 and AAvV-1 viruses (titer = 64 and titer = 32, respectively), although both of these antisera do show reactivity across a number of different viruses. The two-way HI reactivity between the antigen/antiserum pairs of the AAvV-10 reference virus and the AAvV-10-like/Adelie penguin/Antarctica/121/2013 virus does demonstrate that these two viruses share some antigenic similarity; however, it is unclear whether the degree of difference provides sufficient evidence to classify this virus as a separate species from AAvV-10.

**FIG 4:**
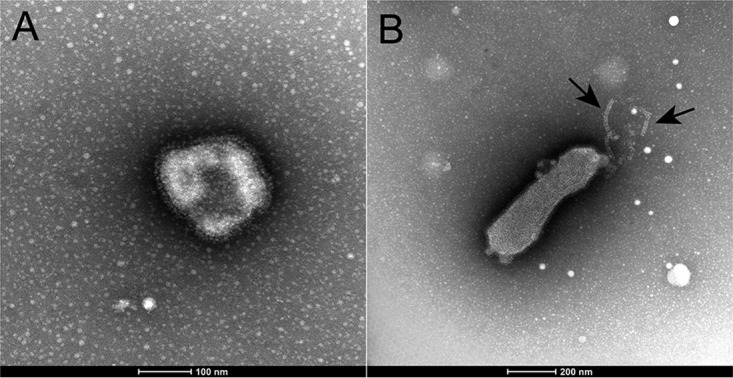
Electron micrographs of AAvV-10-like/Adelie penguin/Antarctica/121/2013 showing spherical (A) and pleomorphic (B) shapes. Herring bone-shaped nucleocapsids are visible, indicated by black arrows in panel B.

Electron microscopy shows that, morphologically, this virus is spherical or pleomorphic in shape, ranging in size from 120 to 200 nm, similar to other AAvVs. Spike-like projections were visible surrounding the envelope (Fig. 4A), as were herring bone-shaped nucleocapsids (Fig. 4B).

### Experimental infection of chickens with novel Antarctic avian avulaviruses.

The pathogenic potential of all four AAvV species was assessed in 4-week-old chickens. No signs of disease were observed in any of the chickens inoculated with any of the Antarctic AAvVs tested, and all chickens survived and remained healthy until the predetermined experimental endpoint (day 21).

**FIG 5:**
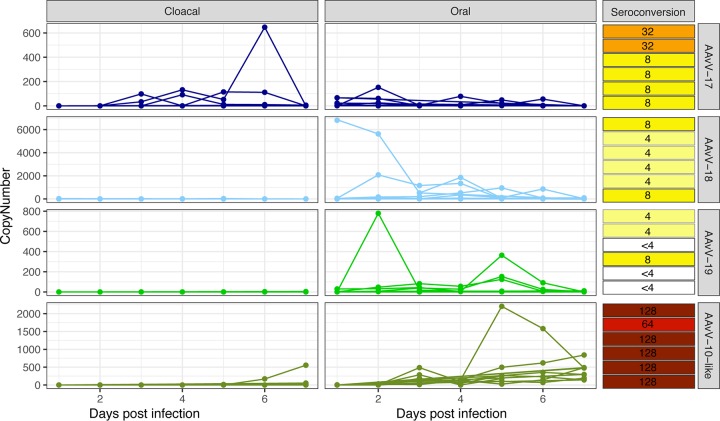
Detection of virus shedding in swab samples collected from chickens inoculated with avian avulaviruses isolated in this study. Viral copy number is presented as number of copies of viral RNA per qRT-PCR reaction mixture volume. Each virus was inoculated into 5 chickens, and oral and cloacal samples collected daily. Chickens in each infection group were shown to be negative for antibodies to the corresponding virus inoculum (HI titers of <4) prior to inoculation. Seroconversion data indicate the postinfection HI antibody titer for each individual chicken in each group at 21 days postinfection and are also presented as a heat map.

To determine levels and patterns of virus shedding, oral and cloacal swab samples were collected over the first 7 days postinoculation (dpi) and tested by reverse transcription-quantitative PCR (qRT-PCR). Virus was detected in all chickens inoculated, with the exception of one chicken (number 16) inoculated with AAvV-18/Adelie penguin/Antarctica/132/2013; however, seroconversion was demonstrated in this chicken, suggestive of low-level infection. Overall, birds shed predominately from the oropharynx, with high copy numbers detected in birds infected with AAvV-18 (peak of 5,625 copies/reaction mixture volume at 2 dpi), AAvV-19 (peak of 779.3 copies/reaction mixture volume at 2 dpi), and AAvV-10-like (peak of 2,205 copies/reaction mixture volume at 5 dpi) and much lower levels of shedding in birds inoculated with AAvV-17 (peak of 152 copies/reaction mixture volume at 3 dpi). Most birds had very limited shedding from the cloaca, where shedding was almost entirely absent prior to 4 dpi. The exception was birds inoculated with AAvV 10-like, where there was a peak of cloacal shedding in one bird at 6 dpi (1,581 copies/reaction mixture volume) (Fig. 5).

Chickens in all infection groups were found to have seroconverted by day 21, with the exception of three birds in the AAvV-19 group, in which HI antibodies were not detected. However, virus shedding was detected in these birds, indicating infection. The highest levels of HI titers were found in birds infected with AAvV-10-like virus (titers of 64 to 128), followed by AAvV-17 (titers of 8 to 32), while low-level HI antibody responses were found in chickens infected with AAvV-18 and -19 (titers of 4 to 8).

## DISCUSSION

Despite its isolation, Antarctica is home to a diversity of fauna and associated microorganisms, including viruses (27), parasites (28), and even antimicrobial-resistant bacteria (29). As such, Antarctic wildlife plays an important role in the epidemiology of many microorganisms. While avian influenza A viruses have only been isolated from Antarctic penguins in recent years (30–32), AAvVs have been isolated and antibodies against these viruses have been detected in Antarctic penguins since the 1980s (5, 14–18, 21), suggesting that penguins may be an important reservoir, rather than spillover hosts, for some AAvV species.

In this study, we identified four different AAvVs in the same penguin species during the same sampling expedition, demonstrating that penguins may be important hosts for a diversity of AAvVs. Three species, AAvV-17, -18, and -19, were recently identified in gentoo penguins on the Antarctic peninsula (5). In our study, AAvV-17 was the most common and had three distinct genetic lineages, and we hypothesize that genetic evolution may have occurred in order to escape host immune responses in reservoir hosts—a pattern well characterized in avian influenza viruses (33). The fourth species, AAvV-10-like, had not been previously identified in Antarctica but, rather, in penguin species of the Falkland Islands and Brazil (20, 24, 25). Unlike AAvV-17, -18, and -19, which were very similar in both our study and that of Neira et al. (5), AAvV-10-like/Adelie penguin/Antarctica/121/2013 is distinct from AAvV-10 viruses isolated in Brazil and the Falkland Islands, potentially suggesting that geography may play an important role in the genetic structure of this virus species; that is, this virus may be limited to Antarctic penguins. Because the genetic distance and antigen relationship between AAvV-10-like/Adelie penguin/Antarctica/121/2013 and AAvV-10 is on the threshold for AAvV classification, it is unclear whether this sample is a novel AAvV species or a divergent member of the AAvV-10 species. Although long-term circulation of these viruses in penguins cannot be demonstrated based on either this study or that of Neira et al. (5), three distinct AAvVs were identified in Antarctic penguins in the 1980s. Unfortunately, these viruses were never analyzed by modern genetic techniques or tested antigenically in an HI assay against ratified species, so they have not been classified according to modern nomenclature (14–17). Whether these old viruses correspond to the AAvVs described in our study and that of Neira et al. (5) is unknown, but the frequency of detection of multiple distinct AAvVs across a number of recent and older studies reinforces the role of penguins as reservoirs for a number of AAvV species.

Experimental infections showed that chickens could become infected with each virus species, as evidenced by seroconversion in all but one bird and virus shedding in all birds (Fig. 5). Virus shedding from oral swabs and limited shedding from cloacal swabs indicated that virus replication occurred in both the upper respiratory and gastrointestinal tracts of infected chickens, although it is unknown whether shedding is predominantly oropharyngeal or cloacal in penguins. Unfortunately, due to the collection of combined oropharyngeal and cloacal swabs, we are unable to speculate as to the major route of shedding in penguins. Taken together, our findings indicate that these novel AAvVs are similar in pathotype to lentogenic AAvV-1 strains. The pathogenicity of the AAvV-10-like isolate is consistent with that of AAvV-10, which had an intracerebral pathogenicity index value of zero in day-old specific-pathogen-free (SPF) chickens and produced no disease or clinical signs in adult chickens inoculated intravenously (20). To our knowledge, our study is the first to assess the infectivity and pathogenicity of AAvV-17, -18, and -19, demonstrating that these viruses have many traits similar to those of lentogenic AAvVs, such as those found in wild birds (e.g., see references 10, 11, and 13). Infectivity in chickens does raise the possibility for infection of species beyond penguins. Currently, AAvV-17, -18, and -19 are restricted to penguins in Antarctica, but AAvV-10 has a much broader spatial scale, with detection in Brazil, the Falkland Islands, and Antarctica. These penguins are unlikely to mix, and therefore, infection in widely distributed potential carrier species, such as the kelp gull (*Larus dominicanus*), should be investigated. A more complete understanding of the broader geographic and host range of these viruses will enable a more accurate assessment of the potential for spread beyond Antarctica.

### Concluding remarks.

Given that many AAvV species have only recently been described and many of these viruses are not economically relevant, the host range and role of different wild bird species as reservoirs for AAvV remains opaque. However, penguins may play an important role in the maintenance of certain AAvV species. Avian avulavirus 1 has a broad host range that extends to penguin species (15, 16, 21, 34). Avian avulaviruses 2, 8, and 10, which are phylogenetically related sister species, have also been isolated in and/or antibodies against these virus species have been detected in Antarctic penguins (20, 23, 35, 47). Avian avulaviruses 17, 18, and 19 are all sister species, suggesting an ancient introduction and, potentially, expansion of AAvV species in penguins. Overall, it is unlikely that penguin AAvV species are clustered together by chance; rather, there appears to be an important geographic, ecological, or host species driver causing these effects. To further disentangle the ecology of AAvVs, further studies of AAvVs in Antarctic penguins and other hosts that share the environment across an annual cycle are warranted.

## MATERIALS AND METHODS

### Ethics statement.

Approval to conduct sampling from penguins in Antarctica was provided by the Universidad de Concepción, Facultad de Ciencias Veterinarias, Chillán, Chile (application number CE-3-2010), and Instituto Antártico Chileno, Chile (application number 03/2013).

Experimental infection of chickens and production of chicken antisera to selected AAvVs was conducted with the approval of the CSIRO Australian Animal Health Laboratory (AAHL) Animal Ethics Committee (permit numbers 1878 and 1811, respectively). All procedures were conducted according to the guidelines of the National Health and Medical Research Council as described in the *Australian Code for the Care and Use of Animals for Scientific Purposes* (36).

### Nomenclature.

Avian paramyxoviruses have recently been renamed avian avulaviruses and are referred to as such throughout the article. Old nomenclature is retained in phylogenetic trees and in the legend to Fig. 3 to honor strain names in GenBank. In order to avoid confusion, we report the viruses identified by Neira et al. (5) using the recently ratified nomenclature. That is, the descriptors “Antarctic Penguin Virus A, B, and C” used by Neira et al. (5) are referred to as avian avulaviruses 17, 18, and 19, respectively (1). Samples 130, 132, and 661 are referred to by the designations AAvV-17/Adelie penguin/Antarctica/130/2013, AAvV-18/Adelie penguin/Antarctica/132/2013, and AAvV-19/chinstrap penguin/Antarctica/661/2013. Virus isolate 121 (which may or may not be a novel viral species) is referred to as AAvV-10-like (AAvV-10-like/Adelie penguin/Antarctica/121/2013).

### Study site.

Adelie penguins were sampled at the following two locations and time periods on the Antarctic peninsula (Fig. 1): adjacent to Arctowski Station, Admiralty Bay, King George Island (62°9′35″S, 58°28′17″W) from 14 to 31 January 2013, and on Kopaitik Island, Rada Covadonga, 1 km west of General Bernardo O’Higgins Base, Antarctic Peninsula (63°19’5”S, 57°53’55”W) from 1 to 15 February 2013. A small number of gentoo penguins and chinstrap penguins were additionally sampled on Kopaitik Island, Rada Covadonga. The penguin colony adjacent to Arctowski Station comprises both Adelie and chinstrap penguins, and a census in 2013 reported 3,246 and 6,123 nests and 3,627 and 6,595 chicks, respectively (37). Isla Kopaitik is a mixed colony containing Adelie, chinstrap, and gentoo penguins, but no survey has been done since 1996 (37). Both colonies are subject to human disturbance.

Samples were collected as described in reference 31. Briefly, a combined oropharyngeal and cloacal sample was collected from each individual and placed in viral transport medium. Samples were kept on ice for up to 4 h before being frozen at −80°C. Cold chain was maintained until samples arrived at the laboratory in Melbourne, Australia, for analysis.

### Screening for avian avulavirus.

RNA was extracted from original swab samples for screening of influenza A virus as described in reference 31. Samples were subsequently assayed using a pan-AAvV RT-PCR assay (38) targeting domain III of the polymerase gene of AAvV-1 to -9 using the SuperScript III first-strand synthesis system (Invitrogen) and AmpliTaq gold 360 DNA polymerase (Applied Biosystems).

All samples were also screened by virus isolation in embryonated chicken eggs. Briefly, original swab samples were diluted 1:1 with phosphate-buffered saline (PBS) containing 1% neomycin-polymyxin solution (bioCSL) and inoculated by the allantoic route into 10-day-old embryonated chicken eggs. Allantoic fluid was harvested after 3 days, and AAvV was detected by hemagglutination with 1% turkey red blood cells, followed by sequence confirmation.

Prevalence estimates across age and location were compared using Fisher’s exact test. A *P* value of <0.05 was taken to indicate a significant difference between compared rates.

### Full-genome sequencing and bioinformatic analysis.

RNA was extracted from the allantoic samples using the QIAamp viral RNA minikit (Qiagen, Valencia, CA, USA) according to the manufacturer’s protocol. An extraction blank was also included during the extraction process. DNA was removed from all RNA samples using Ambion DNA-free (Life Technologies) according to the manufacturer’s protocol. The DNA-free RNA (5 μl) was converted to single-stranded cDNA (Life Technologies) using a sequence-independent RT-PCR primed with 100 μM SIA-1 primer (39, 40) and followed by RNase H digestion. The cDNA was then amplified using a random universal PCR to ensure there was at least 1 μg of DNA for sequencing requirements. Reaction conditions were as follows: 50 μl total reaction mixture volume, including 35 μl of Invitrogen Platinum PCR supermix (Life Technologies), 10 μl of cDNA, 25 mM MgCl_2_, and 100 μM SIA-1 and Extend-1 primers, as described elsewhere (39, 40). The cycling parameters were 2 min at 94°C, followed by 9 cycles of 94°C for 45 s, 25°C for 1 min, and 72°C for 1 min and an additional 50 cycles as follows: 94°C for 45 s, 55°C for 1 min, and 72°C for 1 min. PCR products were visualized by agarose gel electrophoresis, followed by purification using the QIAquick PCR purification kit (Qiagen) and DNA quantitation using the Qubit double-stranded DNA (dsDNA) broad-range (BR) assay (Life Technologies).

DNA libraries were prepared from the purified amplified DNA using the Nextera XT DNA library preparation kit (Illumina, San Diego, CA, USA), followed by sequencing of 150-bp paired-end reads on an Illumina MiSeq instrument. FastQC (http://www.bioinformatics.babraham.ac.uk/projects/fastqc/) was used to check the quality of the Illumina MiSeq sequencing data. Based on the summary report from FastQC, the read length was trimmed after applying an average quality score threshold of 30, and duplicate reads were collapsed using FASTX-Toolkit (version 0.0.13). IVA (Iterative Virus Assembler) (41) was used conduct a *de novo* assembly of the cleaned data. Assembled contigs were validated by mapping the paired-end reads to them using Bowtie2 (42). Consensus sequences were called using SAMTools (43).

### Phylogenetic analysis.

Both individual genes (encoding M, P, N, F, HN, and L proteins [matrix protein, phosphoprotein, nucleoprotein, fusion glycoprotein, hemagglutinin-neuraminidase, and RNA-dependent RNA polymerase, respectively]) and full-genome sequences were aligned using MAFFT within Geneious 10 (Biomatters, New Zealand). Reference viruses included 2 to 5 representatives from each species if possible, in addition to all full-genome viruses from penguins. Maximum-likelihood trees were constructed with 1,000 bootstrap replicates, incorporating the best nucleotide substitution model, using PhyML 3.0 on the ATCG server (http://www.atgc-montpellier.fr/phyml/). Trees were projected using FigTree 1.4.3 (http://tree.bio.ed.ac.uk/software/figtree/).

Nucleotide pairwise distances within and among groups were calculated using MEGA 7.0.14 (44) using the full genomes with the 3′ and 5′ untranslated regions (UTRs) trimmed off. Values for genetic diversity are presented as 1-pairwise distance. Similarly, amino acid pairwise distance was calculated for the L gene (encoding the RNA-dependent RNA polymerase). Cutoffs of <60% nucleotide identity (26) and <80% amino acid identity (45) are the accepted thresholds to distinguish between avian avulavirus species.

### Antigenic characterization.

A hemagglutination inhibition (HI) assay was performed following standard methods (46) and using paired antigens and antisera for AAvV-1 to -12, with the exception of AAvV-5 and -11, for which such combinations were not available. HI titers were expressed as the reciprocal of the highest dilution causing inhibition of 4 hemagglutinin (HA) units of virus.

Isolates AAvV-17/Adelie penguin/Antarctica/130/2013, AAvV-18/Adelie penguin/Antarctica/132/2013, AAvV-19/chinstrap penguin/Antarctica/661/2013, and AAvV-10-like/Adelie penguin/Antarctica/121/2013 were used as representatives of each viral species isolated in this study, and antisera for each of these viruses were raised in chickens as follows. Each virus was propagated in eggs and then inactivated with binary ethyleneimine (BEI). One milliliter of BEI-inactivated virus was added to 2 ml PBS, and then 1.2 ml of the diluted antigen was added to 2.8 ml Montanide V70 adjuvant and the mixture emulsified by homogenization 8 times for 30 s using an Omni mixer homogenizer on speed setting 5, with 1 min of dwell time on ice between each homogenization. Five hundred microliters of this mixture was then subcutaneously inoculated into each chicken. Serum was collected from all chickens 21 days after primary inoculation (prime antisera). For selected birds, booster inoculations of 10^7^ 50% egg infective doses (EID_50_) of virus per bird (0.5 ml) were administered dropwise via the oral, nasal, and ocular routes. Large-volume serum collection was performed on the remaining birds and on boosted birds at 21 days after booster inoculation.

### Experimental infection of chickens.

The pathogenicity of AAvV-17/Adelie penguin/Antarctica/130/2013, AAvV-18/Adelie penguin/Antarctica/132/2013, AAvV-19/chinstrap penguin/Antarctica/661/2013, and AAvV-10-like/Adelie penguin/Antarctica/121/2013 in chickens was assessed by inoculating 0.5-ml volumes containing 10^6^ EID_50_ of each virus into groups of six 4-week-old SPF birds dropwise via the oral, nasal, and ocular routes. Back titrations of each inoculum were performed in embryonated chicken eggs. Following virus inoculation, the oral and cloacal cavities from all chickens were sampled daily for 7 days using sterile cotton swabs placed in 1 ml PBS. Chickens were monitored daily for clinical signs consistent with AAvV disease, including reduced activity, diarrhea, hunched posture, dyspnea, depression, ruffled feathers, and neurological signs. At 21 days postinoculation (dpi), all chickens were humanely euthanized for collection of serum and selected tissues for virologic analysis.

Viral nucleic acids were extracted from all swab samples using the MagMax-96 total RNA isolation kit (Life Technologies). The presence of viral genomes within swab and tissue samples from chickens inoculated with each of the AAvVs was determined using reverse transcription-quantitative PCR (qRT-PCR) assays specific for the polymerase (L) gene of each virus (Table 2). RT-PCR assays were conducted using AgPath-ID one-step RT-PCR reagent (ThermoFisher Scientific) in a total volume of 15 μl, consisting of 7.5 μl of 2× RT-PCR buffer, 0.6 μl of 25× RT-PCR enzyme mix, 0.6 μl of primer and probe mixture (final concentrations, 0.9 μM each primers and 0.25 μM probe) and 5 μl of RNA extract. The thermocycling conditions were 45°C for 10 min, 95°C for 10 min, and 45 cycles of 95°C for 15 s and 60°C for 45 s. Cycle threshold (*C_T_*) values of <45 were considered positive. *C_T_* values for each sample were compared to those obtained for synthetic RNA standards (Integrated DNA Technologies) to calculate the number of gene copies per reaction mixture volume.

**TABLE 2:** Details of primers and probes used to detect avian avulaviruses in specimens collected from chickens inoculated with representative isolates from this study

Virus	Primer/probe^*a*^	Sequence (5′→3′)	Amplicon size (bp)
AAvV-17/Adelie penguin/Antarctica/130/2013	APMV-130F	AGCATACCCAGAGAGTCTTATTCGA	76
	AMPV-130-R	CTCTCACCGTCCTCTGCATACA	
	AMPV-130-Probe	HEX-TGCTCAAATCCCCTCCTCTCCGGT-BHQ1	
AAvV-18/Adelie penguin/Antarctica/132/2013	APMV-132F	CGCAGCGCCACTTTGG	76
	APMV-132R	GGGACCTGGGCCTGGATA	
	AMPV-132-probe	FAM-CCAGCTCCGGTGCAGTTCATCGA-BHQ1	
AAvV-19/chinstrap penguin/Antarctica/661/2013	AMPV-661F	GGCCTCAATCGACGGAATC	105
	AMPV-661R	CGACTGATACCGCCAGTTAGTG	
	APMV-661-Probe	HEX-AAAGCACGAGTTGCCACATTCCTAACCAC-BHQ1	
AAvV-10-like/Adelie penguin/Antarctica/121/2013	APMV-121F	CACTCTGTGCTGACCCTTATGC	83
	APMV-121R	TGCCCGCTGGGTATGTTT	
	APMV-121-Probe	FAM-CTCAACATCCCGTATACTCAGCTCCCCA-BHQ1	

a Primers and probes are specific for the viral polymerase (L) gene.

### Electron microscopy.

Viral isolates AAvV-10-like/Adelie penguin/Antarctica/121/2013 and AAvV-18/Adelie penguin/Antarctica/132/2013 were imaged using electron microscopy. Specimens were negatively stained with 3% phosphotungstic acid (pH 7) on 400-mesh Formvar carbon-coated grids and examined with an FEI Tecnai spirit electron microscope.

### Data availability.

All sequences generated in this study have been deposited in GenBank under accession numbers MK167210 to MK167233.

## Supplementary Material

Supplemental file 1
