# Supplementary material for: Antarctic Penguins as Reservoirs of Diversity for Avian Avulaviruses
Source: J Virol. 2019 May 15;93(11):e00271-19. doi: 10.1128/JVI.00271-19 (PMC6532105; doi:10.1128/JVI.00271-19)
Supplement: Supplemental file 1 [file JVI.00271-19-s0001.pdf]

Antarctic penguins as reservoirs for a diversity of avian avulaviruses

Michelle Wille, Malet Aban, Jing Wang, Nicole Moore, Songhua Shan, John Marshall, Daniel Gonzalez Acuña, Dhanasekaran Vijaykrishna, Jeff Butler, Jianning Wang, Richard Hall, David T. Williams, Aeron C. Hurt

Supplementary Files:
Figure S1-S7

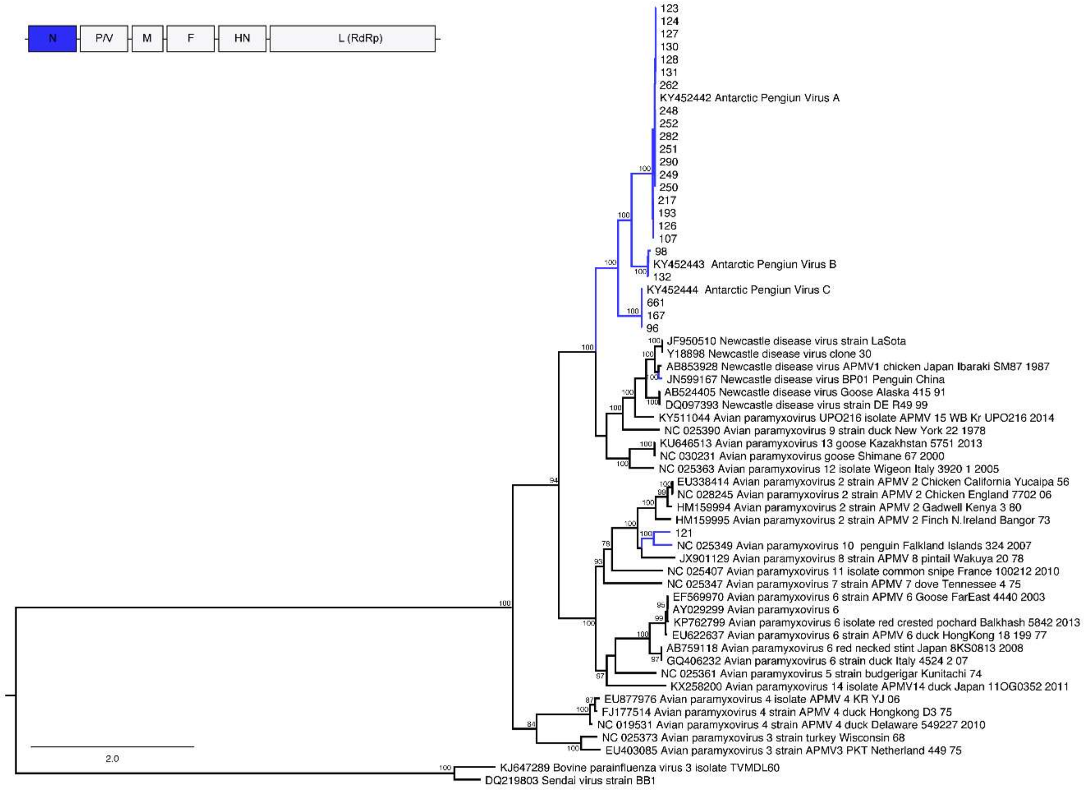

Figure S1. Maximum likelihood tree of the N gene of avian avulaviruses. Outgroup is Bovine Parainfluenza and Sendai Virus. Scale bar represents the number of substitutions per site. Blue branches/clades are those associated with penguins.

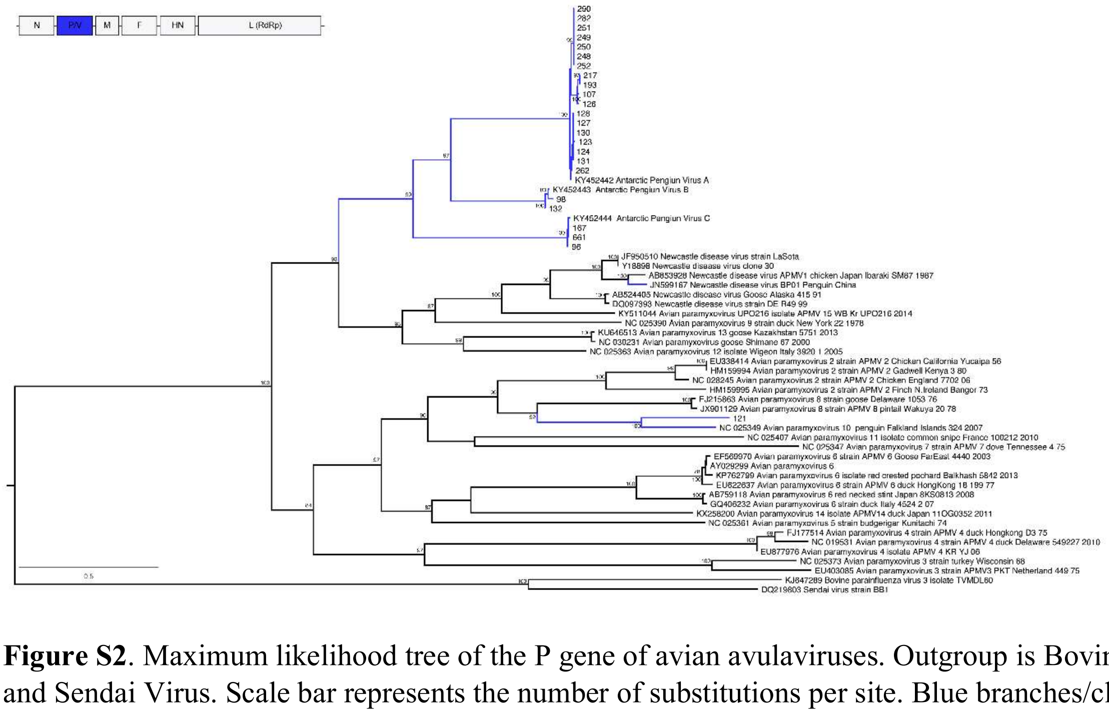

Figure S2. Maximum likelihood tree of the P gene of avian avulaviruses. Outgroup is Bovine Parainfluenza and Sendai Virus. Scale bar represents the number of substitutions per site. Blue branches/clades are those associated with penguins

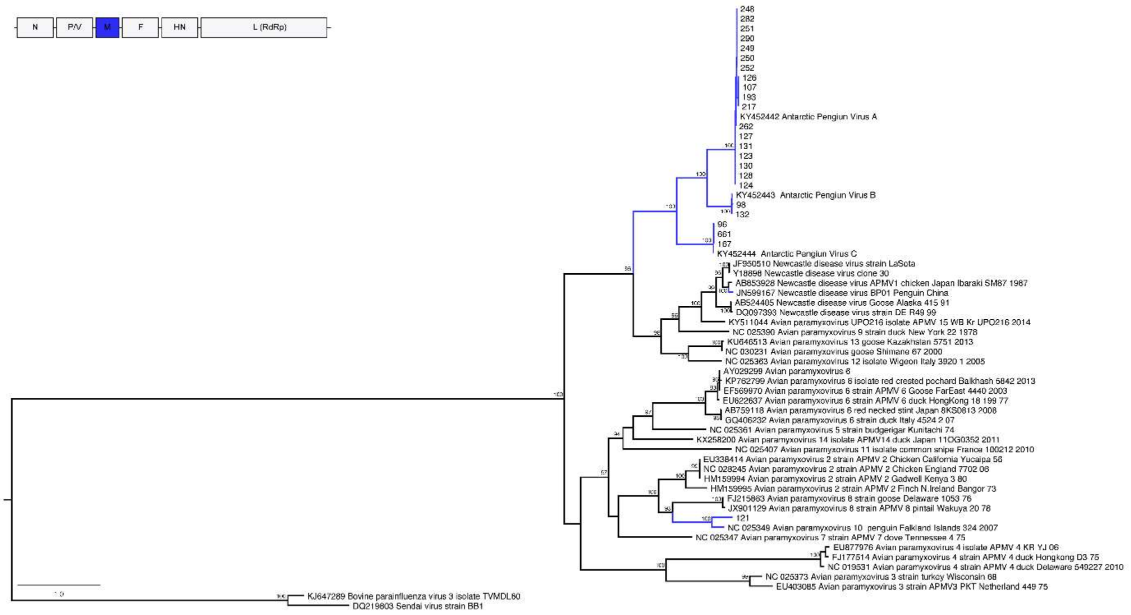

Figure S3. Maximum likelihood tree of the M gene of avian avulaviruses. Outgroup is Bovine Parainfluenza and Sendai Virus. Scale bar represents the number of substitutions per site. Blue branches/clades are those associated with penguins

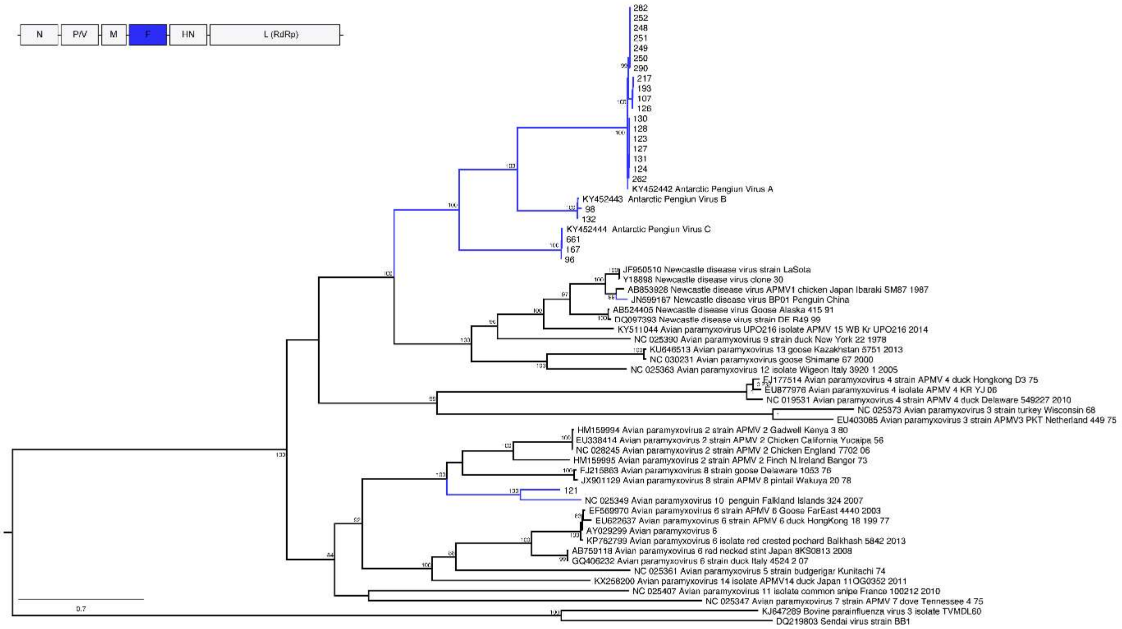

Figure S4. Maximum likelihood tree of the F gene of avian avulaviruses. Outgroup is Bovine Parainfluenza and Sendai Virus. Scale bar represents the number of substitutions per site. Blue branches/clades are those associated with penguins

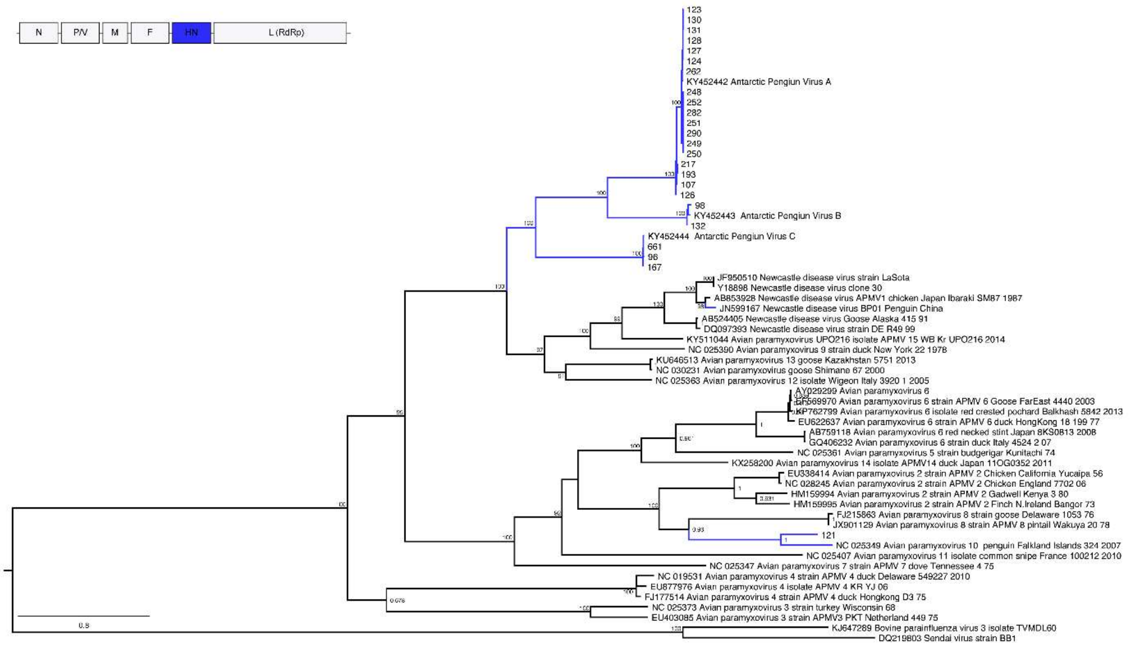

Figure S5. Maximum likelihood tree of the HN gene of avian avulaviruses. Outgroup is Bovine Parainfluenza and Sendai Virus. Scale bar represents the number of substitutions per site. Blue branches/clades are those associated with penguins

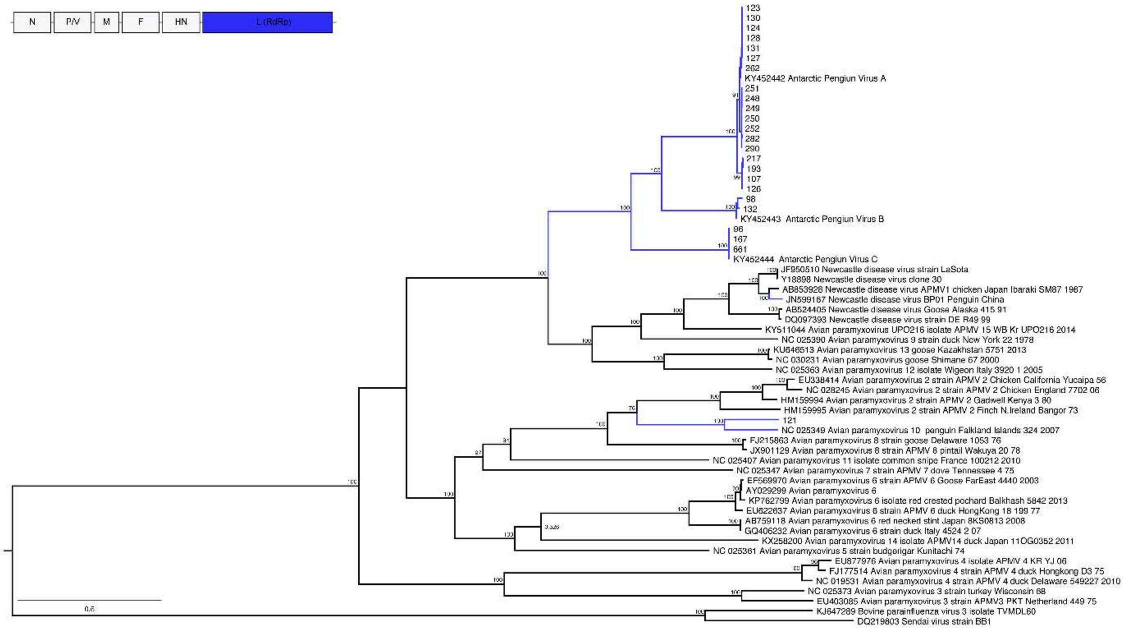

Figure S6. Maximum likelihood tree of the L gene of avian avulaviruses. Outgroup is Bovine Parainfluenza and Sendai Virus. Scale bar represents the number of substitutions per site. Blue branches/clades are those associated with penguins

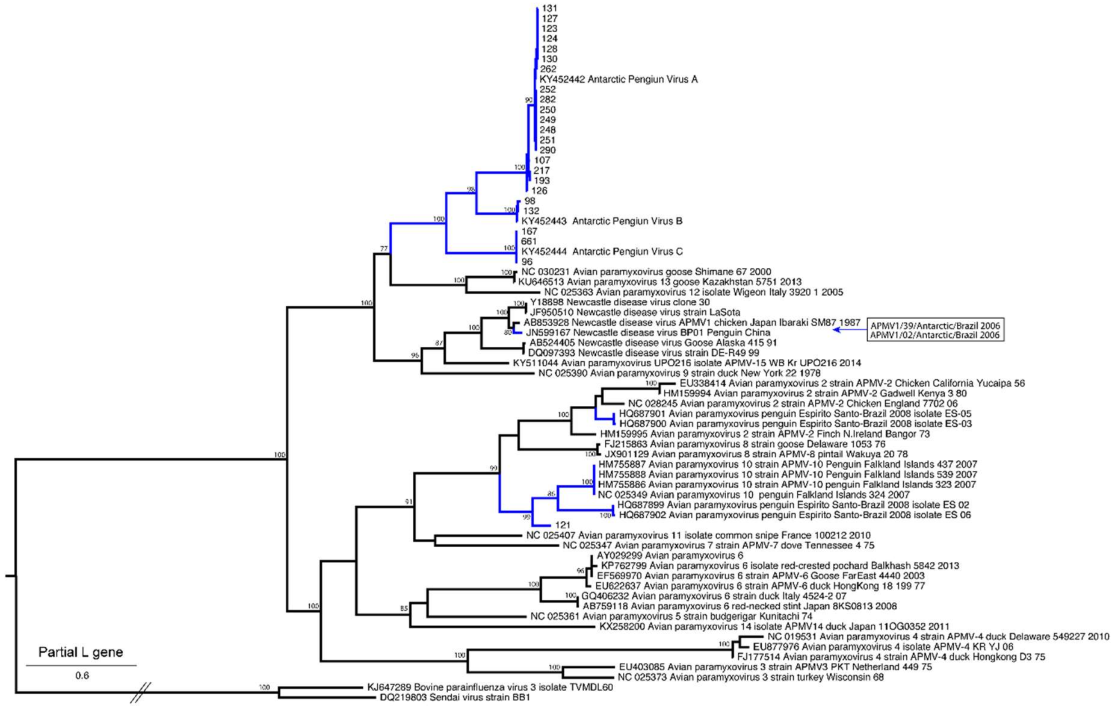

Figure S7. Maximum likelihood of partial L gene of avian avulaviruses. Utilization of partial L gene allows for the inclusion of AAvV-10 and AAvV -2 from penguins in Brazil. Approximate position of AAvV -1 from Antarctica are shown, but not included as available sequences are from the F gene. Outgroup is Bovine Parainfluenza and Sendai Virus. Scale bar represents the number of substitutions per site. Blue branches/clades are those associated with penguin.
